# Supplementary material for: Identification of dysregulated genes in rheumatoid arthritis based on bioinformatics analysis
Source: PeerJ. 2017 Mar 15;5:e3078. doi: 10.7717/peerj.3078 (PMC5356478; doi:10.7717/peerj.3078)
Supplement: Table S1 — DEGs, differentially expressed genes; FDR, false discovery rate; NA, not available. [file peerj-05-3078-s001.docx]

**Table S1 The top 10 up-/down-regulated DEGs and those DEGs interacted with top 10 up-/down-regulated DEGs enriched in GO terms**

| **Items** | | **Items detail** | **Gene**  **count** | **FDR** | **top 10 up-regulated**  **DEGs enriched in it** | **top 10 down-regulated DEGs enriched in it** | **DEGs interacted with top 10 up/down-regulated DEGs** |
| --- | --- | --- | --- | --- | --- | --- | --- |
| **Biolocial process** | | | | | | | |
| GO:0008380 | RNA splicing | | 19 | 9.89E-08 | RBFOX2 | NA | THOC2,DDX5 |
| GO:0007165 | Signal transduction | | 35 | 2.39E-05 | TNK1 | ATF6B | NPM1,ESR2,PRKAG1 |
| GO:0010467 | Gene expression | | 19 | 3.43E-05 | EIF5B | TARS,NR5A1 | ESR2 |
| **Molecular function** | | | | | | | |
| GO:0005515 | Protein binding | | 162 | 4.77E-48 | RBFOX2,SERBP1,TNK1,  CD47,WIPF1,LCK,EIF5B | SIK3,TCOF1,NR5A1 | THOC2,NPM1,TOP1,DDX5,  RPA3,NOP56,ATXN2,ESR2,PIAS1,  ATN1,RPA2,WASL,PRKAG1 |
| GO:0000166 | Nucleotide binding | | 66 | 3.56E-13 | RBFOX2,SAR1A,TNK1,  PFKFB3,LCK,EIF5B | TARS,SIK3,MAT2A | RAB2A,TOP1,DDX5,PRKAG1 |
| GO:0005524 | ATP binding | | 45 | 3.29E-08 | RBFOX2,SAR1A,TNK1,  PFKFB3,LCK,EIF5B | TARS,SIK3,MAT2A | RAB2A,TOP1,DDX5,PRKAG1 |

DEGs: differentially expressed genes; FDR: false discovery rate; NA: not available.
