# Supplementary material for: Identification of dysregulated genes in rheumatoid arthritis based on bioinformatics analysis
Source: PeerJ. 2017 Mar 15;5:e3078. doi: 10.7717/peerj.3078 (PMC5356478; doi:10.7717/peerj.3078)
Supplement: Table S2 — NA: not applicable; DEGs: differentially expressed genes; RA: rheumatoid arthritis. The predicted proteins of TRD, POU6F1 and CA5A were not available in the BioGRID database and were not shown in Table S2. [file peerj-05-3078-s002.docx]

**Table S2 The DEGs interacted with the top 10 up- and down-regulated DEGs in RA**

| **Gene ID** | **Gene symbol** | **Up/down in RA** | **DEGs interacted with it in PPI network**  **[Gene ID(gene symbol, up/down in RA)]** |
| --- | --- | --- | --- |
| **top 10 up-regulated genes in RA** | | | |
| 3932 | LCK | up | 8976(WASL, up) |
| 26135 | SERBP1 | up | 8554(PIAS1,up) |
| 8711 | TNK1 | up | 1655(DDX5,up) |
| 7456 | WIPF1 | up | 8976(WASL, up), |
| 5209 | PFKFB3 | up | NA |
| 23543 | RBFOX2 | up | 1655(DDX5,up),1822(ATN1,down),2100(ESR2,down), 6118(RPA2,up),6119(RPA3,up),6311(ATXN2,down), |
| 56681 | SAR1A | up | NA |
| 961 | CD47 | up | NA |
| 9669 | EIF5B | up | NA |
| **top 10 down-regulated genes in RA** | | | |
| 4144 | MAT2A | down | NA |
| 1388 | ATF6B | down | 3133(HLA-E, up), 5862(RAB2A,up), |
| 26148 | C10orf12 | down | NA |
| 6949 | TCOF1 | down | 6118(RPA2,up),6119(RPA3,up), 7150(TOP1,down), 10528(NOP56,down) |
| 2516 | NR5A1 | down | 57187(THOC2,down) |
| 6897 | TARS | down | 5571(PRKAG1,up), |
| 10412 | NSA2 | down | 4869(NPM1,up) |
| 23387 | SIK3 | down | NA |

NA: not applicable; DEGs: differentially expressed genes; RA: rheumatoid arthritis. The predicted proteins of TRD, POU6F1 and CA5A were not available in the BioGRID database and were not shown in Table S2.
